# Fibrinogen Binding Sites P_336_ and Y_338_ of Clumping Factor A Are Crucial for *Staphylococcus aureus* Virulence

**DOI:** 10.1371/journal.pone.0002206

**Published:** 2008-05-21

**Authors:** Elisabet Josefsson, Judy Higgins, Timothy J. Foster, Andrej Tarkowski

**Affiliations:** 1 Department of Rheumatology and Inflammation Research, University of Gothenburg, Göteborg, Sweden; 2 Microbiology Department, Moyne Institute of Preventive Medicine, Trinity College, Dublin, Ireland; University of Birmingham, United Kingdom

## Abstract

We have earlier shown that clumping factor A (ClfA), a fibrinogen binding surface protein of *Staphylococcus aureus*, is an important virulence factor in septic arthritis. When two amino acids in the ClfA molecule, P_336_ and Y_338_, were changed to serine and alanine, respectively, the fibrinogen binding property was lost. ClfAP_336_Y_338_ mutants have been constructed in two virulent *S. aureus* strains Newman and LS-1. The aim of this study was to analyze if these two amino acids which are vital for the fibrinogen binding of ClfA are of importance for the ability of *S. aureus* to generate disease. Septic arthritis or sepsis were induced in mice by intravenous inoculation of bacteria. The *clfA*P_336_Y_338_ mutant induced significantly less arthritis than the wild type strain, both with respect to severity and frequency. The mutant infected mice developed also a much milder systemic inflammation, measured as lower mortality, weight loss, bacterial growth in kidneys and lower IL-6 levels. The data were verified with a second mutant where *clfA*P_336_ and Y_338_ were changed to alanine and serine respectively. When sepsis was induced by a larger bacterial inoculum, the *clfA*P_336_Y_338_ mutants induced significantly less septic death. Importantly, immunization with the recombinant A domain of ClfAP_336_SY_338_A mutant but not with recombinant ClfA, protected against septic death. Our data strongly suggest that the fibrinogen binding activity of ClfA is crucial for the ability of *S. aureus* to provoke disease manifestations, and that the vaccine potential of recombinant ClfA is improved by removing its ability to bind fibrinogen.

## Introduction

Clumping factor A (ClfA) is a surface located protein of *Staphylococcus aureus*. ClfA contains a 519 amino acid N-terminal A domain, which comprises three separately folded subdomains N1, N2 and N3. The A domain is followed by a serine-aspartate dipeptide repeat region and a cell wall- and membrane-spanning region, which contains the LPDTG-motif for sortase-promoted anchoring to the cell wall. ClfA is present in practically all *S. aureus* strains [1]. It binds to the C-terminus of the γ-chain of fibrinogen, and is thereby able to induce clumping of bacteria in fibrinogen solution [2], [3].

Expression of ClfA on *S. aureus* hampers phagocytosis by both macrophages and neutrophils [4], [5]. In neutrophils this is due to both a fibrinogen-dependent mechanism and to a fibrinogen-independent mechanism. In contrast, platelets are activated by bacteria expressing ClfA through its interaction with GPIIb/IIIa leading to aggregation. This is most efficiently executed when fibrinogen is present, but there is also a fibrinogen-independent pathway for platelet activation [6], [7].

We have earlier shown that ClfA is a virulence factor for induction of septic arthritis in mice [8]. Also, elimination of ClfA together with another fibrinogen binding protein ClfB protected against systemic inflammation at the early stages of infection [9].

It is not known how expression of ClfA contributes to the development of arthritis and sepsis. Especially, the role of the interaction between ClfA and host fibrinogen in the pathogenesis of severe staphylococcal infections is presently unclear. In this study the role of fibrinogen binding of ClfA in promoting the virulence of *S. aureus* was investigated in depth.

A fibrinogen binding-deficient mutant of ClfA was constructed by exchanging amino acids P_336_ and Y_338_ for serine and aspartate, respectively. This mutant protein did not promote detectable bacterial adherence to immobilized fibrinogen or bacterial clumping in a solution of fibrinogen [6]. The conformation of single ClfA P_336_S and ClfAY_338_A mutants were unaltered according to far-UV circular dichroism spectroscopy [10]. The wild-type chromosomal *clfA* genes of strains Newman and LS1 were exchanged for the *clfA* P_336_SY_338_A mutant and the strains were tested in well established infection models of murine septic arthritis and sepsis [11], [12]. In addition a deletion mutant lacking ClfA expression was compared to the wildtype and non-fibrinogen binding ClfA expressing mutant. Our data strongly suggest that interaction between ClfA and fibrinogen is crucial for infection morbidity and mortality, and points out a possibility to create a more efficient approach to vaccination.

## Results

### Exchange of two amino acids necessary for ClfA binding to fibrinogen hampers development of septic arthritis and sepsis

Two amino acids (P336 and Y338) that are known to be required for fibrinogen binding by ClfA were altered by allelic exchange to create mutants of strains Newman and LS1 that expressed a non-fibrinogen-binding ClfA protein on the cell surface. The level of expression and integrity of the protein was measured by Western blotting.

**Figure 1:**
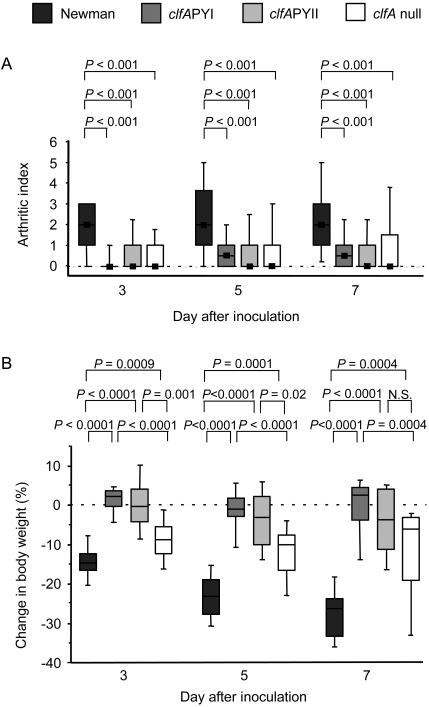
The fibrinogen binding site of ClfA mediates development of septic arthritis and loss of weight. Severity of arthritis (A), measured as arthritic index, and weight loss (B) in mice inoculated with *S. aureus* strain Newman, and *clfA*PYI, *clfA*PYII, and *clfA* null mutants. 3.2×10^6^–6.0×10^6^ cfu of *S. aureus* strains were inoculated. Data are represented as medians (squares or center lines), interquartile ranges (boxes), and 80% central ranges (whiskers). Data are from three experiments are pooled. *N*_Newman_ = 27–30, *N_clfA_*_PYI_ = 30, *N_clfA_*_PYII_ = 10, and *N_clfA_* = 16–20.

The ability of Newman wild-type and Newman *clfA* P_336_S Y_338_A (*clfA*PYI) to provoke septic arthritis was investigated. Septic arthritis was induced by intravenous inoculation of 3.5×10^6^ to 5.0×10^6^ colony-forming units (cfu) and 3.2×10^6^ to 6.0×10^6^ cfu of Newman wild-type and the *clfA*PYI mutant, respectively. The development of arthritis was studied clinically for 7 days. The *clfA*PYI mutant provoked significantly less severe arthritis than the wild-type strain over the entire experimental period (*P*>0.001, Fig. 1 A). The frequency of arthritis was lower for Newman *clfA*PYI at all time points (Fig. S1).

We reasoned that the new amino acid composition in the ClfAPYI molecule accidentally fits for interaction with a host anti-bacterial defence which could be a possible explanation for our data. To check for this possibility, a new construct was made where different amino acids were substituted for P336 and Y338 (*clfA* P_336_A Y_338_S : *clfA*PYII). Mice that were inoculated with 3.9×10^6^ cfu of Newman *clfA*PYII developed arthritis to the same low extent as the *clfA*PYI mutant (Fig. 1 A), and with a similar frequency (Fig. S1). This outcome suggests strongly that the loss of fibrinogen binding is responsible for the reduced level of arthritis.

It is possible that ClfA is involved in the development of arthritis by mechanisms that do not involve fibrinogen binding. A *clfA* deletion mutant lacking the ClfA protein was compared to mutants expressing the modified non-fibrinogen binding ClfA protein. However, mice that were infected with 4.3×10^6^ to 4.8×10^6^ cfu of *clfA* null mutant developed arthritis in a manner not different from the *clfA*PYI and *clfA*PYII mutant infected mice (Fig. 1 A). The frequency of arthritis was also indistinguishable (Fig. S1).

Infected joints were also investigated histologically. The synovitis in Newman *clfA*PYI-infected mice was significantly milder than in wild-type infected mice in both experiment 1 and 2 (*P* = 0.02 and 0.001, respectively, Table 1). Bone destruction, a major cause of sequels in human septic arthritis, was almost absent in the Newman *clfA*PYI-infected samples (Experiment 2, *P* = 0.001, Table 1). The synovitis and bone destruction induced by the Newman *clfA* null mutant were also less pronounced compared to mice infected with Newman wild-type (*P* = 0.003 and 0.006, respectively, Table 1), but somewhat more severe than in the Newman *clfA*PYI group, although not significantly so.

**Table 1:** Histologic joint affection after i.v. inoculation with *S. aureus* strain Newman, *clfA*PYI mutant and *clfA* null mutant.

			Synovitis^a^		Cartilage/bone destruction^a^	
Experiment	Strain	No. of mice	Index^b^	Frequency, %	Index^b^	Frequency, %
Experiment 1	Newman	7	10.0 (7.7, 22.3)	86	6.0 (1.1, 13.7)	86
	*clfA*PYI mutant	10	4.8 (1.3, 6.5)^c^	80	1.1 (0, 3.0)	70
Experiment 2	Newman	9	15.0 (8.8, 15.6)	100	8.0 (3.5, 9.5)	100
	*clfA*PYI mutant	10	0.6 (0, 2.7)^d^	50	0 (0, 1.3)^d^	50
	*clfA* null mutant	8	2.3 (0.6, 4.2)^e^	75	1.3 (0, 2.1)^f^	63

a The severity and frequency of synovitis and cartilage/bone destruction was monitored in mice 7–8 days after bacterial i.v. inoculation.

b Data are median of histologic index (lower, upper quartiles).

c *P* = 0.02 versus wild-type.

d *P* = 0.001 versus wild-type.

e *P* = 0.003 versus wild-type.

f *P* = 0.006 versus WT.

Next, the metabolic consequences of the *clfA* mutations for the infectious process were analysed. Mice infected with the Newman wild-type strain lost up to about 30% of their body weight during the experimental period. Mice that were infected with the fibrinogen binding-deficient mutants Newman *clfA*PYI and Newman *clfA*PYII lost hardly any weight at all (*P*>0.0001 versus wild-type). In contrast, the Newman *clfA* null mutant had an intermediate effect on the weight loss, causing significantly less than the wild-type strain, but significantly more than the *clfA*PYI and *clfA*PYII mutant strains (*P* ≤ 0.02 in most cases, Fig. 1 B).

The serum levels of IL-6, a measure of systemic inflammatory response, were analyzed at day 7–8 of infection. The pattern of IL-6 expression was similar to weight changes. Newman wild-type evoked high levels of serum IL-6 (4.8 (2.8, 5.7) ng/ml), the Newman *clfA*PYI mutant evoked considerably lower IL-6 (0.2 (0.07, 2.4) ng/ml, *P*<0.0001) while the Newman *clfA* null mutant gave rise to an intermediate response (2.5 (1.3, 3.2) ng/ml) with significant differences to both the wild-type and *clfA*PYI mutant group (*P* = 0.009 and *P* = 0.008, respectively) (median, interquartile range).

**Figure 2:**
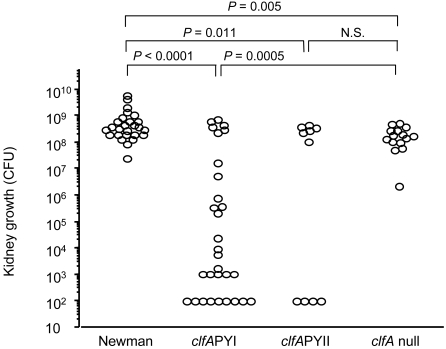
Bacterial growth in kidneys is hampered upon mutation of the ClfA fibrinogen binding site. Bacterial growth in kidneys in mice 7–8 days after inoculation with 3.2×10^6^–6.0×10^6^ cfu of *S. aureus* strain Newman, and *clfA*PYI, *clfA*PYII, and *clfA* null mutants. Data are represented as cfu per kidney pair. One circle represents the kidney counts of one mouse. Where no growth was detectable, the count was put to highest possible count according to what dilution was used. Data from three experiments are pooled. *N*_Newman_ = 26, *N_clfA_*_PYI_ = 30, *N_clfA_*_PYII_ = 10, and *N_clfA_* = 15.

The growth of bacteria in kidneys was significantly greater in Newman wild-type-infected mice, compared to both of the Newman *clfA*PY mutants and the Newman *clfA* null mutant (*P*<0.0001, *P* = 0.011, and *P* = 0.005, respectively; Figure 2). The Newman *clfA* null mutant-infected mice had significantly more bacterial growth in kidneys than Newman *clfA*PYI-infected mice (*P* = 0.0005, Fig. 2).

Total IgG in sera was measured in mice on day 7–8 of infection. There was a significantly lower increase of IgG levels in both the Newman *clfA*PYI- and Newman *clfA* null mutant-infected groups as compared to mice infected with the wild-type strain (3.1 (1.2, 4.9); 2.3 (1.0, 2.6); and 6.4 (5.0, 11.0), respectively (median, interquartile range); *P* ≤ 0.0003). There were no significant differences between the two mutant groups.

The mortality was 17% in the Newman wild type-infected mice, 0% in the Newman *clfA*PYI and *clfA*PYII mutant groups and 30% in the Newman *clfA* null mutant group. There were significant differences in mortality between the wild-type and the *clfA*PYI groups, and between the *clfA*PYI and *clfA* null mutant groups (*P*<0.05 and *P*<0.01, respectively).

We conclude that direct and indirect signs of systemic inflammation are lower in mice infected with *S.aureus* expressing ClfA that is deficient in fibrinogen binding. Unexpectedly, the strain which lacked ClfA expression altogether induced more systemic inflammation than a ClfAPY mutant-expressing strain. There could be activities mediated by ClfA that do not involve fibrinogen binding but which are protective to the host with respect to systemic inflammation.

**Figure 3:**
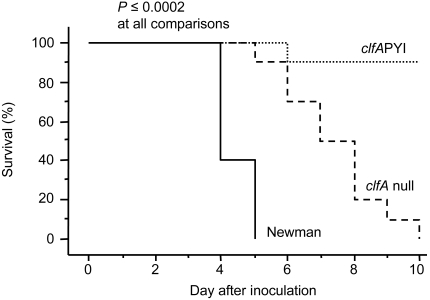
The ClfA fibrinogen binding site affects survival after strain Newman challenge. Survival of mice after inoculation with 5.2, 5.1 or 3.3×10^7^ cfu of *S.aureus* strain Newman, *clfA*PYI mutant or *clfA* null mutant, respectively. *N* = 10 per group from start.

Sepsis was induced in mice by increasing the inoculation dose of *S. aureus*. Mice were infected with 5.2×10^7^ cfu of Newman wild type, 5.1×10^7^ cfu of the Newman *clfA*PYI mutant and 3.3×10^7^ cfu of the Newman *clfA* null mutant. Within 5 days all wild-type infected mice were dead, but only one *clfA*PYI mutant mouse out of ten were dead after 10 days of infection (*P*<0.0001, Fig. 3). Mice infected with the *clfA* null mutant also survived a significantly shorter time than the *clfA*PYI mutant- infected mice (*P*<0.0001, Fig. 3). In this experiment the mice challenged with the *clfA* null mutant developed significantly more arthritis than the *clfA*PYI mutant group, while at the same time they lost significantly more weight (Fig. S2 and S3). Thus, by analogy with the measures of systemic inflammation in the septic arthritis experiments, the survival of the mice is prolonged if the ClfA molecule is expressed, as long as it lacks fibrinogen binding properties.

### Injection of bacteria into joints

To test if the inflammatory reaction in the joint is dependent on fibrinogen binding, Newman wild-type, Newman *clfA*PYI or Newman *clfA* null were injected directly into a knee joint of mice, thereby by-passing the systemic compartment. Synovitis, including polymorphonuclear infiltration of the joint cavity, and bone destruction was studied by histology 3 days later. The mice received 2.4×10^4^ cfu of wild-type, 2.4×10^4^ cfu of the *clfA* null mutant, or 3.4×10^4^ cfu of *clfA*PYI mutant in one knee. The synovitis and the polymorphonuclear infiltration histologic index in the joint cavity was 0.25 (0, 3.0) for knees infected with wild-type, 2.38 (0.25, 3.0) for the *clfA* null mutant and 0.25 (0, 0.25) for the *clfA*PYI mutant (median, interquartile range). The histologic index for destruction of bone was 0 (0, 1.0) for wild-type, 1.0 (0, 1.0) for the *clfA* null mutant, and 0 (0, 0) for the *clfA*PYI mutant (median, interquartile range; *P* = 0.01 between the *clfA*PYI mutant and the *clfA* null mutant). Since the *clfA*PYI mutant evoked very little synovitis and destruction, despite the fact that 42% more of that strain was given to mice than the other strains, it is concluded that ClfA-promoted fibrinogen binding is needed for the maximal inflammatory response within the joint. Again, the absence of ClfA expression enhanced inflammation compared to the fibrinogen binding deficient ClfA mutant.

### PY mutation in strain LS-1

**Figure 4:**
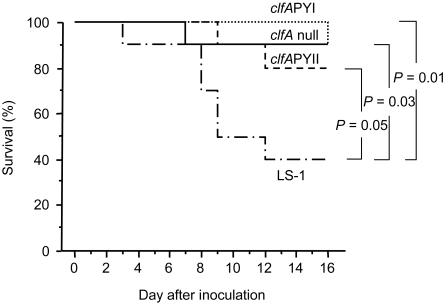
The ClfA fibrinogen binding site affects virulence of strain LS-1. Survival of mice after inoculation with 9.4, 7.9, 10.7 or 9.8×10^6^ cfu of *S.aureus* strain LS-1, and *clfA*PYI, *clfA*PYII or *clfA* null mutants, respectively. *N* = 15 per group from start.

To determine if the ability of ClfA to bind fibrinogen affects virulence of other strains of *S.aureus*, the *clfA*PYI, *clfA*PYII and *clfA* null mutations were transduced to the TSST-1 expressing *S. aureus* strain LS-1. Mice were challenged with 9.4×10^6^ cfu of LS-1 wild-type, 7.9×10^6^ cfu of LS-1 *clfA*PYI , 10.7×10^6^ cfu of LS-1 *clfA*PYII, or 9.4×10^6^ cfu of the LS-1 *clfA* null mutant. Sepsis was studied by following the survival rate. After 16 days only 40% of mice challenged with the wild-type strain were alive while 90% of the mice challenged with the *clfA*PYI mutant and *clfA* null mutant groups and 80% mice infected with the *clfA*PYII mutant were alive (Fig. 4). The *clfA*PYI mutants and the *clfA* null mutant of LS-1 were significantly less virulent (*P* = 0.014, *P* = 0.05 and *P* = 0.03, respectively).

### Immunization with recombinant ClfA proteins

**Figure 5:**
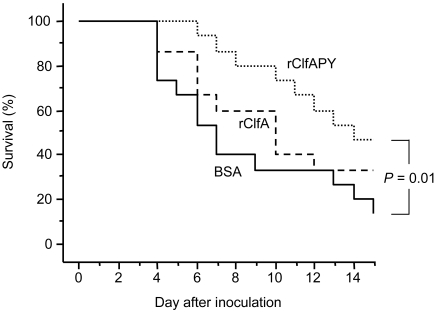
Vaccination efficacy of ClfA is improved by mutation of the fibrinogen binding site. Survival of mice immunized with BSA, recombinant ClfA or recombinant ClfAPY and inoculated with 2.3×10^7^ cfu of *S. aureus* Newman. *N* = 15 per group from start.

The effect of vaccination with recombinant wild-type ClfA A domain protein (rClfA) and mutant ClfAPYI protein (rClfAPY) was studied in both the septic arthritis model and the sepsis model. Mice were sensitized and then boosted twice with control protein BSA, rClfA, or rClfAPY, and subsequently infected with 4.0×10^6^ cfu of *S. aureus* strain Newman to induce septic arthritis, or with 2.3×10^7^ cfu of strain Newman to induce sepsis. Immunization with rClfAPY protected significantly against septic death as compared to control mice (*P* = 0.01, Fig. 5) while rClfA immunization did not achieve significant protection. One day before bacterial infection there was a much higher specific serum antibody response to both rClfAPY and rClfA in mice immunized with rClfAPY (A_405_ = 0.39 (0.33, 0.56) and 0.71 (0.52, 0.81)) as compared to mice immunized with rClfA (A_405_ = 0.13 (0.07, 0.17) and 0.15 (0.10, 0.24), *P*<0.0001 in both comparisons (median, interquartile range)). Control immunized animals had only background levels (*A*_405 nm_ = 0 and 0.01 (0, 0.01) (median, interquartile range)). The immunized mice which were to be infected with the lower, arthritic bacterial dose had similar antibody responses to rClfA and rClfAPY as the mice in which sepsis were induced (data not shown). Immunization with both rClfA and rClfAPY protected against the development of arthritis, although the protection was not significant (Fig. S4). During day 5 to 9 after infection the weight loss was significantly reduced in the rClfAPY and rClfA immunized mice, as compared to the control mice (data not shown). A trend to diminished bacterial growth in kidneys of mice immunized with rClfAPY or rClfA at day 11 after infection (BSA: 38 (3, 436); rClfAPY: 7 (2, 17); rClfA: 10 (7, 54)×10^7^ cfu/kidney pair) was observed.

## Discussion

The impact of the fibrinogen binding of ClfA on the ability of *S. aureus* to evoke septic arthritis and sepsis was investigated. Our data strongly suggest that the ClfA-fibrinogen interaction is crucial for the bacterial virulence and thus disease outcome. The ability of ClfA to bind fibrinogen was associated with enhanced virulence in terms of the ability to cause septic death. In both staphylococcal strains tested, a *clfA*PY mutant induced less septic death than the wild-type. Also, the severity of arthritis was strongly reduced in mice infected with the non-fibrinogen binding *clfA*PY mutant.

A likely mechanism for the promotion of virulence by the fibrinogen-bacterial cell surface interaction is inhibition of neutrophil phagocytosis. Opsonophagocytosis experiments with human neutrophils show clearly that ClfA inhibits phagocytosis of *S. aureus*, and that this in part is dependent on fibrinogen binding because the ClfAPY mutant is only partially inhibiting [5]. Neutrophils are crucial for the host defence in the early phase of *S. aureus* infection [13]. Without neutrophils, bacterial growth is strongly augmented in blood and kidneys, and the frequency of arthritis and mortality increases. Fibrinogen mediated inhibition of neutrophil phagocytosis by ClfA could explain at least in part the more pronounced virulence of wildtype *S. aureus* compared to the *clfA*PY mutants. Binding of fibrinogen to ClfA could decrease opsonophagocytosis by neutrophils by reducing opsonin deposition or access to opsonins by neutrophil receptors. ClfA could also specifically block phagocytosis, for example by interfering with the fibrinogen binding phagocyte integrin Mac-1. The streptococcal M5 protein has been shown to block phagocytosis by neutrophils by interfering with Mac-1 [14], and the fibrinogen binding region of the M5 protein is essential for resistance to phagocytosis [15]. Alternatively, bound fibrinogen might block the binding of an unknown protective host factor to *S. aureus*. Another option is that the fibrinogen-ClfA interaction promotes bacterial passage from blood vessel into the tissue or promotes colonization in tissues.

Unexpectedly, our data also show the ClfA null mutant was more virulent than the *clfA*PY mutant strains. Possibly the ClfA protein has functions in vivo other than interacting with fibrinogen. This interaction is clearly disadvantageous for the host as shown in this study. Other functions of ClfA are presently not well mapped but non-fibrinogen dependent platelet aggregation exerted by ClfA might result in trapping of big amounts of *S. aureus* in circulation with subsequent elimination of the bacterial–platelet complexes through the reticuloendothelial system. Such platelet aggregation mediated elimination of staphylococci would readily occur in the wild-type and *clfA*PY mutated strains but not in the *clfA* knockout. Whereas in the wild-type strain the fibrinogen interaction would overshadow the other events, in the *clfA*PY mutants such bacterial eliminiation might be highly beneficial to the host.

The *clfA* knockout mutant protected against septic death to the same degree as the *clfA*PY mutation in *S. aureus* strain LS-1, but protected less, if at all, in strain Newman. The overall impact of ClfA expression on bacterial virulence could differ between different *S. aureus* strains depending on the level of expression and the presence of other virulence factors.

The issue whether the *clfA*PY mutant displays equal or lower virulence once in the joint cavity is of certain importance having in mind that in inflamed synovial fluid fibrinogen and fibrin are abundant. Our data suggest that the *clfA*PY mutant is less destructive for cartilage and bone.

The protective effect of recombinant ClfA A domain non-fibrinogen binding P_336_Y_338_ mutant was greater than for wild-type rClfA. Immunization with ClfAPY enhanced the serum levels of specific antibodies that recognized both the immunogen and the wild-type ClfA protein. More importantly, it induced a greater protective immune response against septic death than wild-type ClfA. In contrast, we did not see a similar significant correlation in case of septic arthritis. We interpret the differences between the two infection models to accessibility of antibodies to different body compartments which is excellent in the blood stream (sepsis) and lower in the joint cavity during septic arthritis.

We hypothesize that binding of fibrinogen by wild-type ClfA protein during the immunization phase decreases antigen presentation due to hiding of important epitopes on the ClfA molecule and hence decreases specific antibody production. This finding implies that PY mutant ClfA might be a considerably better vaccine candidate than wild type recombinant ClfA.

## Materials and Methods

### Mice

NMRI mice were obtained from Scanbur BK (Sollentuna, Sweden) and were maintained in the animal facility of the Department of Rheumatology, University of Göteborg, Sweden. Göteborg animal experiment ethic board approved the experiments, and all experiments conform to the animal husbandry standards of this ethic board. The mice were housed up to 10 animals per cage with a 12 h light-dark cycle, and were fed standard laboratory chow and water ad libitum. The animals were 6 to 16 weeks old at the start of the experiments.

### Bacterial strains

For infection of animals the *S. aureus* wildtype strains Newman [16] and LS-1 [11] and constructed derivatives thereof were used. The *clfA* P_336_SY_338_A (*clfA*PYI) and *clfA* P_336_AY_338_S (*clfA*PYII) derivatives were constructed in strain Newman and transduced to strain LS-1 (see below). The deletion mutants Newman *clfA2::Tn917* mutant DU5876 [3] and LS-1 *clfA2::Tn917* mutant (J.R. Fitzgerald et al., unpublished) were also used. Bacteria were grown on blood agar plates for 48 h, harvested, and kept frozen at −20°C in PBS containing 5% (wt/vol) BSA (Sigma Chemicals) and 10% (vol/vol) dimethyl sulfoxide. Before injection into animals, the bacterial suspensions were thawed, washed in PBS, and adjusted to appropriate cell concentrations. The number of viable bacteria was measured in conjunction with each challenge by cultivation on blood agar plates and counting colonies.

### Construction of *clfA*PYI and *clfA*PYII mutations in *S. aureus* Newman and LS-1

A 1.02 kb *Pst*I-*Bam*HI fragment of pCF77 PY [6] containing the mutations P_336_S and Y_338_A in *clfA* was cloned into pBluescriptII SK- (Stratagene). This plasmid was linearised with *Hind*III and ligated to *Hind*III-cut pTS*ermC* (J. Higgins et al., unpublished) to generate plasmid pARM, which is a temperature sensitive *E. coli*-*S. aureus* shuttle vector containing the P_336_S and Y_338_A substitutions.

In order to reduce the risk of unknowingly generating a functional or immunoreactive epitope by substituting P_336_ and Y_338_, we generated a second mutant, in which the order of the substitutions was reversed, yielding P_336_A and Y_338_S. To generate this a plasmid pJH2, analogous to pARM but containing the P_336_A and Y_338_S subsitutions, was generated. Overlap primer PCR was used with the same flanking primers used to make pCF77 PY [6], and a different pair of overlapping mutagenic primers: F3: GCAACTTTGACCATG**G**CCGCTT**C**TATTGACCCTGAAAATG and R3: CATTTTCAGGGTCAATA**G**AAGCGG**C**CATGGTCAAAGTTGC (mutations in bold and underlined) to generate pCF77 PYII. The 1.02 kb PstI-HindIII fragment of this plasmid was used as described above to generate pJH2, a temperature sensitive *E. coli*-*S. aureus* shuttle vector containing the P_336_A and Y_338_S substitutions.

Both pARM and pJH2 were transferred to RN4220 [17] by electroporation and subsequently transduced using phage 85 [18] to *S. aureus* Newman [16] and LS-1 [11]. In these strains the plasmids were induced to insert into the chromosome and then excise, leaving the mutations in the chromosome of a proportion of transformants, generating Newman *clfA*PYI, Newman *clfA*PYII, LS-1 *clfA*PYI and LS-1 *clfA*PYII. Transformants were screened for loss of the plasmid and a loss of fibrinogen-binding activity. Integrity of the *clfA* gene was verified by Southern hybridisation using a *clfA* probe (data not shown). Expression of an immunoreactive protein (ClfAPY) was verified by Western immunoblotting using anti-ClfA region A polyclonal rabbit antiserum (data not shown). The mutations were verified by PCR across the *Kpn*I-*Bam*HI fragments from genomic DNA and commercial sequencing of the products. The about 700 bases of the *clfA* gene of strain LS-1 that were sequenced were identical to the corresponding bases in the Newman *clfA* gene of strain Newman.

### Recombinant ClfA and ClfAPY

His-tagged recombinant ClfA region A, domains N123 (amino acids 40–559), was produced from pCF40 as previously described [19], with an additional polishing step through an anion-exchange column. Plasmid pCF77 PY [6] was used as template to clone *clfA*PYI domains N123 into pQE30 to generate pCF40PY. Using this plasmid, recombinant ClfAPY was also produced by nickel affinity chromatography and anion exchange chromatograpy, as was described for rClfA. Eluates were dialysed against two changes of PBS before concentration and freeze-drying.

### Septic arthritis and sepsis experiments

In experiments 1–3 all the mice (n = 10 per group) were infected with strain Newman to trigger arthritis. In experiments 4 and 5, the mice were infected with strain Newman and LS-1, respectively, to induce sepsis (n = 10 per group).

#### Experiment 1

Mice were infected by intravenous injection with 3.5×10^6^ cfu/mouse of *S.aureus* strain Newman or with 4.3×10^6^ cfu/mouse of Newman *clfA*PYI mutant, both in 200 μl PBS. Clinical arthritis and weight change was followed until day 7. Mice were sacrificed day 8, kidney growth of bacteria were assessed and serum IL-6 and total IgG levels were measured. Synovitis and bone destruction was studied histologically on the joints of fore and hind legs.

#### Experiment 2

Mice were infected with 5.0×10^6^ cfu, 6.0×10^6^ cfu or 4.3×10^6^ cfu of *S.aureus* strain Newman, *clfA*PYI mutant or Newman *clfA::Erm^R^* (*clfA* null mutant), respectively. Clinical arthritis and weight change was followed until day 7. Mice were sacrificed day 7, kidney growth of bacteria were assessed and serum IL-6 and total IgG levels were measured. Synovitis and bone destruction was studied histologically on the joints of fore and hind legs.

#### Experiment 3

Mice were infected with 4.7×10^6^ cfu, 3.2×10^6^ cfu, 3.9×10^6^ cfu or 4.8×10^6^ cfu of *S.aureus* strain Newman, *clfA*PYI mutant, Newman *clfA*PYII mutant or Newman *clfA* null mutant, respectively. Clinical arthritis and weight change was followed until day 7. Mice were sacrificed day 8 and kidney growth of bacteria were assessed.

The outcome of the experiments 1–3 were very similar, so data were pooled and presented together.

In experiment 4 mice were injected intravenously with 5.2×10^7^ cfu, 5.1×10^7^ cfu or 3.3×10^7^ cfu of *S.aureus* strain Newman, *clfA*PYI mutant or *clfA* null mutant, respectively. Mortality, weight change and clinical arthritis were followed until day 10.

In experiment 5 mice were infected with 9.4×10^6^ cfu, 7.9×10^6^ cfu, 10.7×10^6^ cfu or 9.8×10^6^ cfu of *S.aureus* strain LS-1, LS-1 *clfA*PYI mutant, LS-1 *clfA*PYII mutant, or LS-1 *clfA* null mutant, respectively. Mortality, clinical arthritis and weight change was followed until day 16.

### Intra-articular injection of bacteria

One knee joint per mouse was injected with 2.4×10^4^ cfu, 2.4×10^4^ cfu, or 3.4×10^4^ cfu of strain Newman wildtype, *clfA*PYI mutant or *clfA* knockout mutant, respectively, in 20 μl PBS. N = 10 per group. Mice were sacrificed 3 days later, and the knee joints were collected for histopathological examination.

### Vaccination with wild-type and mutant recombinant ClfA

Purified rClfA, rClfAPY or BSA were dissolved in physiologic saline and emulsified 1∶1 in Freund́s complete adjuvant (Difco Laboratories). Two hundred μl of the emulsion containing 30 μg of protein was injected subcutaneously (s.c.) on day 0. First booster immunization with 30 μg of protein in physiologic saline in incomplete Freund́s adjuvant was performed on day 11. Second booster immunization was done day 21.

On day 31, 14–15 mice per group were infected by i.v. injection of 4.0×10^6^ cfu/mouse for induction of septic arthritis, or by 2.3×10^7^ cfu/mouse for induction of sepsis. Clinical arthritis, weight change and mortality were followed for 11 and 15 days, respectively. Bacterial growth in kidneys was assessed in the septic arthritis experiment.

### Clinical evaluation of infected mice

The clinical evaluation was performed in a blinded manner. Each limb was inspected visually. The inspection yielded a score of 0 to 3 (0, no swelling and erythema; 1, mild swelling and/or erythema; 2, moderate swelling and/or erythema; 3 marked swelling and/or erythema). The arthritic index of an animal was constructed by adding the scores from all four limbs. The presented arthritic index is thus the severity of arthritis, measured as the sum of scores, of all mice in a group. The overall condition of each mouse was also examined by assessing signs of systemic inflammation, i.e., weight decrease, reduced alertness, and ruffled coat. In cases of severe systemic infection, when a mouse was judged too ill to survive another 24 h, it was killed by cervical dislocation and considered dead due to sepsis.

### Histological examination

Histological examination of joints was performed using a modification [8] of a previously described method [20]. Briefly, microscopic inspection yielded a score for each joint for severity of synovitis and cartilage/bone destruction. Synovitis was scored as 0–3 and cartilage and/or bone destruction was scored as 0–2. Totally 12 joints per animal were examined. The histologic index, for severity of synovitis and cartilage/bone destruction respectively, was calculated by adding the scores for the 12 joints of an animal.

### Bacteriologic examination of infected kidneys

Kidneys were aseptically dissected, kept on ice, homogenised, serially diluted in PBS and spread on blood agar plates. After 24 h of incubation in 37°C the number of cfu per kidney pair was determined.

### Measurement of serum IgG

Levels in serum of total IgG were measured by the radial immunodiffusion technique [21]. Goat-Anti-Mouse-IgG and mouse IgG standard were purchased from Southern Biotech, Birmingham, AL.

### Specific antibodies

Serum samples from immunized mice were obtained 9 days after the second booster immunization. The serum levels of specific antibodies against rClfA and rClfAPY was measured by ELISA. Microplates (96-well; Nunc) were coated with 5 μg/ml of recombinant protein in PBS. Blocking agent, serum samples, biotinylated antibodies, and ExtrAvidin-proxidase were all diluted in PBS. The assay was run according to a previous description [8]. All serum samples were diluted 1∶20000, and antibody response was monitored as absorbance at 405 nm.

### IL-6 analysis

Serum IL-6 was detected by a method previously described [22].

### Statistical analysis

Statistical evaluation was done by using the Mann-Whitney U test when comparing two groups, the Kruskal-Wallis test with a following post-hoc analysis when there were three or more groups to compare, or the Kaplan-Meier analysis with a Tarone-Ware test at survival analysis. The Chi-square test was used for frequency comparisons. *P*<0.05 was considered to be significant. No correction for multiple comparisons was employed. Data are reported as medians, interquartile ranges, and 80% central ranges, unless otherwise mentioned.

## Supporting Information

Figure S1 The fibrinogen binding site of ClfA mediates enhanced frequency of septic arthritis. Frequency of arthritic mice inoculated with 3.2×10^6^–6.0×10^6^ cfu of *S. aureus* strain Newman wild-type, and *clfA*PYI, *clfA*PYII, and *clfA* null mutants. Data from three experiments are pooled. *N*_Newman_ = 27–30, *N_clfA_*_PYI_ = 30, *N_clfA_*_PYII_ = 10, and *N_clfA null_* = 16–20.
(1.23 MB TIF)

Figure S2 Severity of arthritis in septic mice is ameliorated upon fibrinogen binding site mutation. Severity of arthritis measured as arthritic index in mice inoculated with 5.2, 5.1 or 3.3×107 cfu of *S.aureus* strain Newman wild-type, *clfA*PYI mutant or *clfA* null mutant, respectively. Data are presented as medians (squares), interquartile ranges (boxes), and 80% central ranges (whiskers). *N*_Newman_ = 0–10, *N_clfA_*_PYI_ = 9–10, and *N_clfA null_* = 1–10. All Newman wild-type infected mice were dead by day 5.
(0.42 MB TIF)

Figure S3 Weight loss in septic mice is less pronounced upon fibrinogen binding site mutation. Weight loss in mice inoculated with 5.2, 5.1 or 3.3×10^7^ cfu of *S.aureus* strain Newman wild-type, *clfA*PYI mutant or *clfA* null mutant, respectively. Data are presented as medians (center line), interquartile ranges (boxes), and 80% central ranges (whiskers). *N*_Newman_ = 0–10, *N_clfA_*_PYI_ = 9–10, and *N_clfA null_* = 1–10. All Newman wild-type infected mice were dead by day 5.
(0.42 MB TIF)

Figure S4 Vaccination efficacy of ClfA on septic arthritis with and without mutation of the fibrinogen binding site. Severity of arthritis measured as arthritic index in mice immunized with BSA, recombinant ClfA or recombinant ClfAPY and inoculated with 4.0×10^6^ cfu of *S. aureus* Newman. Data are presented as medians (squares), interquartile ranges (boxes), and 80% central ranges (whiskers). *N*_BSA_ = 14, *N_rclfA_*_PY_ = 14, and *N_rclfA_* = 15 per group from start.
(0.42 MB TIF)
