# Supplementary figures and images for: Fibrinogen Binding Sites P336 and Y338 of Clumping Factor A Are Crucial for Staphylococcus aureus Virulence
Source: PLoS One. 2008 May 21;3(5):e2206. doi: 10.1371/journal.pone.0002206 (PMC2374910; doi:10.1371/journal.pone.0002206)

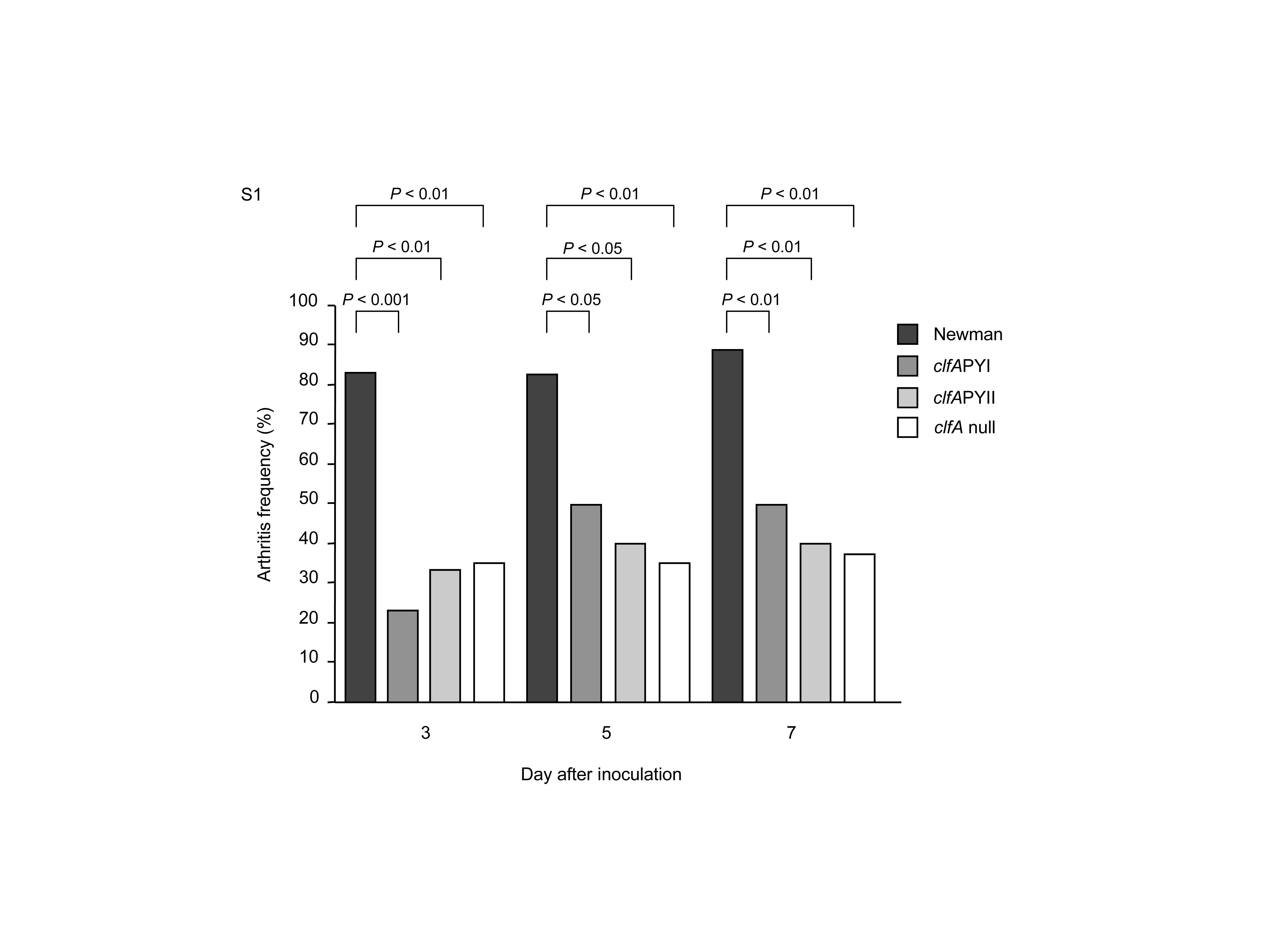

Supplement: Figure S1 — The fibrinogen binding site of ClfA mediates enhanced frequency of septic arthritis. Frequency of arthritic mice inoculated with 3.2×10^6^–6.0×10^6^ cfu of S. aureus strain Newman wild-type, and clfAPYI, clfAPYII, and clfA null mutants. Data from three experiments are pooled. N Newman = 27–30, NclfA PYI = 30, NclfA PYII = 10, and NclfA null = 16–20. (1.23 MB TIF) [file pone.0002206.s001.tif]

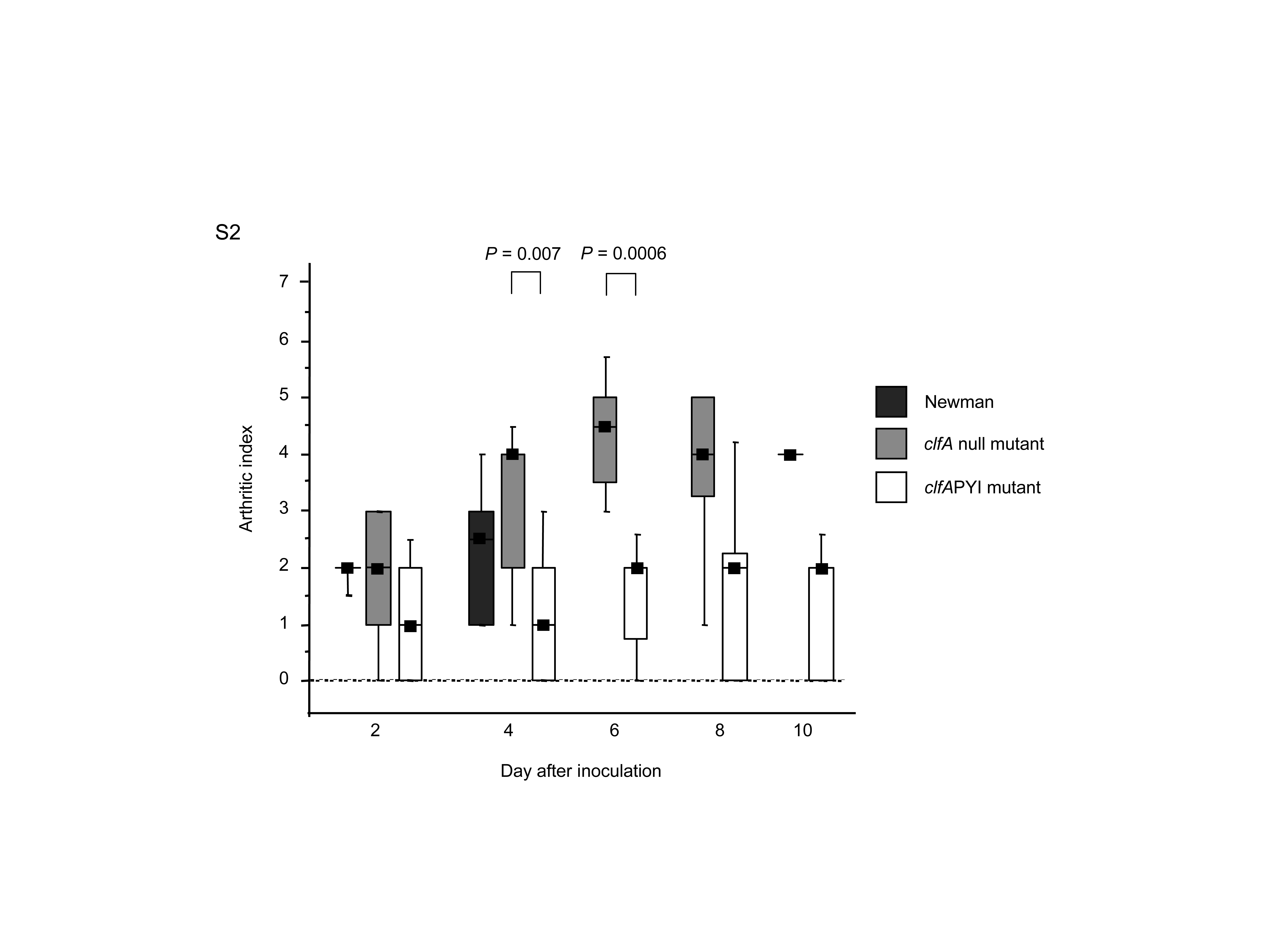

Supplement: Figure S2 — Severity of arthritis in septic mice is ameliorated upon fibrinogen binding site mutation. Severity of arthritis measured as arthritic index in mice inoculated with 5.2, 5.1 or 3.3×10^7^ cfu of S.aureus strain Newman wild-type, clfAPYI mutant or clfA null mutant, respectively. Data are presented as medians (squares), interquartile ranges (boxes), and 80% central ranges (whiskers). N Newman = 0–10, NclfA PYI = 9–10, and NclfA null = 1–10. All Newman wild-type infected mice were dead by day 5. (0.42 MB TIF) [file pone.0002206.s002.tif]

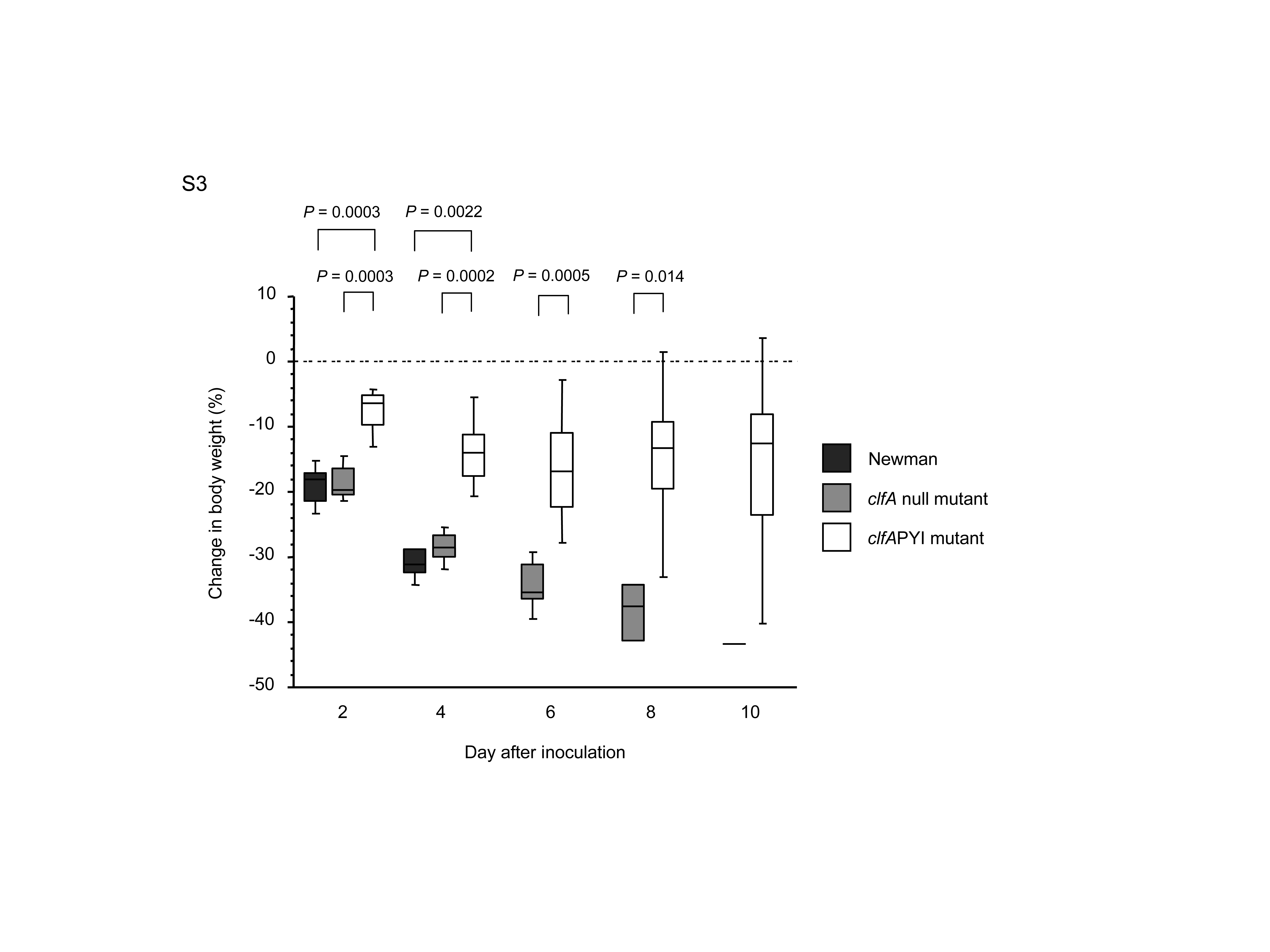

Supplement: Figure S3 — Weight loss in septic mice is less pronounced upon fibrinogen binding site mutation. Weight loss in mice inoculated with 5.2, 5.1 or 3.3×10^7^ cfu of S.aureus strain Newman wild-type, clfAPYI mutant or clfA null mutant, respectively. Data are presented as medians (center line), interquartile ranges (boxes), and 80% central ranges (whiskers). N Newman = 0–10, NclfA PYI = 9–10, and NclfA null = 1–10. All Newman wild-type infected mice were dead by day 5. (0.42 MB TIF) [file pone.0002206.s003.tif]

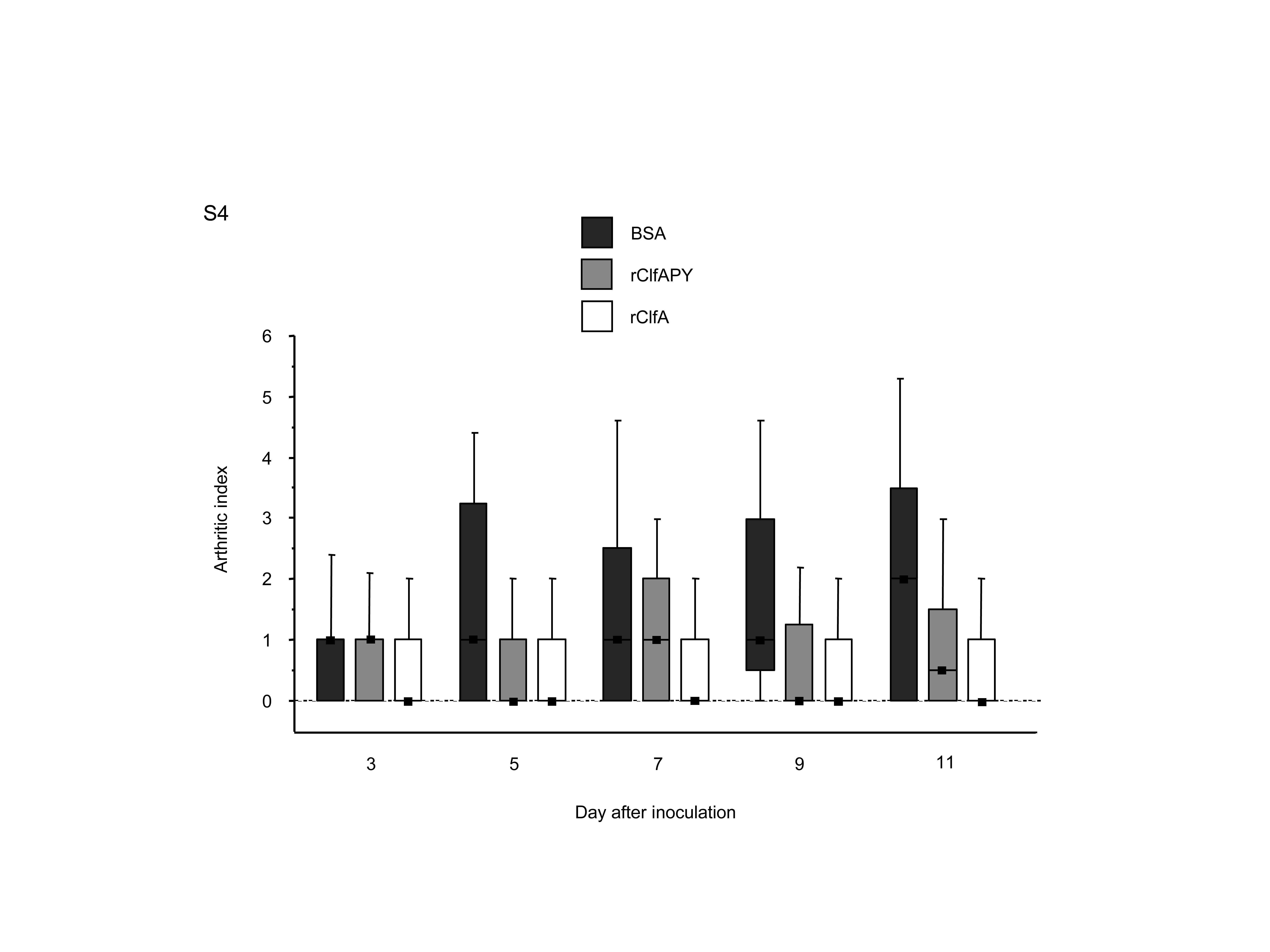

Supplement: Figure S4 — Vaccination efficacy of ClfA on septic arthritis with and without mutation of the fibrinogen binding site. Severity of arthritis measured as arthritic index in mice immunized with BSA, recombinant ClfA or recombinant ClfAPY and inoculated with 4.0×10^6^ cfu of S. aureus Newman. Data are presented as medians (squares), interquartile ranges (boxes), and 80% central ranges (whiskers). N BSA = 14, NrclfA PY = 14, and NrclfA = 15 per group from start. (0.42 MB TIF) [file pone.0002206.s004.tif]
